# Environmental Aesthetic Value Influences the Intention for Moral Behavior: Changes in Behavioral Moral Judgment

**DOI:** 10.3390/ijerph18126477

**Published:** 2021-06-15

**Authors:** Chenjing Wu, Xianyou He

**Affiliations:** 1 School of Psychology, South China Normal University, Guangzhou 510631, China; wuchenjing@m.scnu.edu.cn; 2 Key Laboratory of Brain, Cognition and Education Sciences, Ministry of Education, South China Normal University, Guangzhou 510631, China; 3 Center for Studies of Psychological Application, South China Normal University, Guangzhou 510631, China; 4 Guangdong Key Laboratory of Mental Health and Cognitive Science, South China Normal University, Guangzhou 510631, China

**Keywords:** environmental aesthetic value, moral behavior intention, immoral behavior intention, moral judgment

## Abstract

The environment affects moral behavior. Previous research found that a beautiful environment leads to pro-social behavior, which is related to behavioral intention. However, the effect of environmental aesthetic value on immoral and moral behavior remains unclear. Therefore, in the present study, we explored the effect of environmental aesthetic value on behavioral intention and its possible mechanisms. We conducted four experiments. Experiment 1 adopted the priming paradigm and IAT paradigm to explore the relationship between environmental aesthetic value and behavioral intention. It used photographs of the environment as priming stimuli and scene drawings of behavior as target stimuli. The results showed that participants had a higher intention to engage in moral behavior in an environment with a high aesthetic value, and a lower intention to engage in immoral behavior, compared to in an environment with a low aesthetic value. Similarly, an environment with a low aesthetic value was related to immoral behavior. Experiment 2 further explored the possible mechanism for the above results: changes in moral judgment. The results showed that moral judgment in different environments may lead to different behavioral intentions. The current study extends prior research by demonstrating the effect of environmental aesthetic value on behavioral intention and moral judgment, and good knowledge about the relationship between environmental aesthetic value and moral behavior. In addition, it provides a new hypothesis for the relationship between environment and behavior according to the results of the environment–behavior matching hypothesis, which can provide a new perspective on moral education.

## 1. Introduction

The environment plays an important role in behaviors [1,2,3,4,5]. For example, the environment influences pro-social, generous, and moral behaviors, and it is well known that these are important factors of a good society. Previous research found that a bright environment, one with many plants, and with a clean smell, influences participants to increase positive behaviors [5,6,7,8]. Similarly, prior research has also found that the environment may have a negative effect: disorganization, fewer plants, low brightness, and extreme environmental temperatures may cause violent crimes [2], rule-breaking [9], cheating [10]. In general, the environment plays an important role in daily life.

Aestheticians share similar views regarding the important effect of the environment on behaviors, suggesting that a beautiful environment can positively influence moral behavior. Aesthetics is a psychological state of emotional pleasure caused by the properties of an object. It defines the characteristics of beauty as a pleasurable experience [11]. Moral behavior refers to social behaviors that are adopted under the domination of a certain moral consciousness. This includes moral and immoral behavior. The former refers to positive behaviors, such as being pro-social and helpful. The latter refers to unlawful, morally unacceptable, or dishonest behavior [12]. Zhang et al. (2014) found that a beautiful environment triggers more pro-social behavior [13]. Moral intention is an individual’s subjective determination to act in a moral or immoral manner. Researchers have found a link between intention and behavior, by which 30% of the variance in behavior could be explained by behavioral intention [14,15]. If individuals lack behavioral intentions, they will perform no action. However, there is a lack of relevant experimental research to explore whether an environmental aesthetic value affects intention for moral and immoral behaviors, and how an environmental aesthetic value affects behavioral intention. Experiment 1, therefore, aimed to explore the influence of an environmental aesthetic value on behavioral intention for moral and immoral behaviors.

Moral judgment refers to an individual’s ability to judge the level and degree of moral justification of an action in a situation. Rest (1983) proposed a four-component model of morality by emphasizing the connection between internal changes in cognition and external behavior [16]. Moral judgment is an important component that determines the implementation of moral behavior intentions [17]. It is positively related to moral behavior intention [17,18,19,20]. Chui, Kouchaki, and Gino (2021) found a mediation effect of the acceptability of cheating (moral judgment) on the relationship between larger competitions and cheating behavior [21]. Additionally, some studies have demonstrated that physical environments affect moral judgment [3,22,23,24,25]. For example, Schnall, Haidt, Clore, and Jordan (2008), who explored the environmental role of moral judgment, found that after being exposed to a disgusting environment, participants increased their unfavorable attitudes toward immoral issues [24]. The researchers even found that disgust affects the moral judgment of neutral situations [26,27]. The theory of reasoned action (TRA) also suggests that an individual’s behavior can be reasonably inferred, to some extent, from behavioral intentions, which are determined by attitudes and subjective norms toward behavior. Individuals are rational and consider the meaning and consequences of their behavior by integrating various pieces of information when they adopt a certain behavior [28]. Furthermore, in aesthetic studies, people tend to assign very positive characteristics to beauty.

The phrase “Beauty is good,” for example, suggests that people perceive highly attractive faces to be more kind, intelligent, honest, enthusiastic, and to possess favorable personality traits [29,30]. Studies also found that aesthetics influence individual emotional states: a high aesthetic value (beauty) leads to positive emotions, and a low aesthetic value leads to negative emotions. Emotions influence moral judgments; for example, positive emotions can lead individuals to make a tolerant moral judgment [31]. However, some studies found an inverse relationship between positive emotion and tolerant moral judgment [32]. Similarly, a negative emotion leads participants to judge moral violations (and even neutral events) as more immoral [3,26,27,33,34]. Overall, aesthetic value influences individuals’ moral judgments.

**Figure 1:**
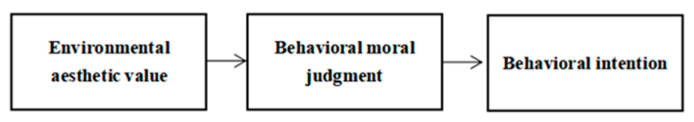
The path showing the influence of environmental aesthetic value on behavioral intention.

Therefore, we proposed a mechanism in which the influence of an environmental aesthetic value on moral intention to act is due to a change in behavioral moral judgment for the effect of environments with different aesthetic values on behavioral intention (see Figure 1). Hence, Experiment 2 investigated and tested whether the effect of an environmental aesthetic value on behavioral intention is related to changes in moral judgment. This would help understand the knowledge of the influence mechanism regarding the effect of an environmental aesthetic value on behavioral intention.

### The Present Research

We conducted four experiments to explore the relationship between the environmental aesthetic value and behavioral intention for moral and immoral behaviors, and the possible reasons for whether moral judgment changes under environments with different aesthetic conditions. We used explicit measurements (the priming paradigm) and implicit measurements (IAT) in Experiment 1 to explore this effect. In the present research, an environment with a high aesthetic value implied a beautiful environment, and an environment with a low aesthetic value indicated a not-beautiful environment. We hypothesized that an environment with a high aesthetic value would trigger higher moral behavior intention, compared to an environment with a low aesthetic value, which leads to a higher intention to engage in immoral behavior. Experiment 2 further tested the possible mechanisms of this effect in Experiment 1. In Experiment 2, we explored the effect of environments with different aesthetic values on moral judgment and the relationship between behavioral intention and moral judgment in such environments, to explain the effect of environmental aesthetic value on behavioral intention from the perspective of a changing moral judgment. Drawing from the predicted results of Experiment 1, Experiment 2 hypothesized that an environment with a high aesthetic value would lead to harsh moral judgment of immoral behavior and lenient moral judgment for moral behavior compared to an environment with a low aesthetic value. In other words, an environment with a low aesthetic value would lead to harsher judgments of moral behavior and tolerant judgments for immoral behavior, compared to an environment with a high aesthetic value.

## 2. Experiment 1

### 2.1. Experiment 1a

To explore the effects of environmental aesthetic value on behavioral intention for moral and immoral behaviors, Experiment 1a chose the behaviors of different moral styles as the target and used environmental photographs with high and low aesthetic values as the primary stimuli to explore whether the aesthetic value affects behavioral intention.

#### 2.1.1. Method

##### Design

Experiment 1a was a 2 (types of environmental aesthetic value: high vs. low) × 2 (types of behavioral scene drawings: moral vs. immoral) within-subject experimental design. Participants were required to evaluate behavioral intention for the same behaviors under the same environmental photographs. The dependent variables were behavioral intention rating scores.

##### Participants

A total of 25 college students aged 18–30 years (17 females; *M* age = 21.16 years, *SD* = 0.98) were recruited and compensated for their participation. We used G * Power 3.1 to estimate the power (1-*β* = 0.42) and effect size (d = 0.48). All participants had normal or corrected normal vision and normal color vision. Additionally, they signed an informed consent form. The protocol was approved by the Ethics Committee of South China Normal University (SCNU-PSY-2020-4-050).

##### Materials

**Figure 2:**
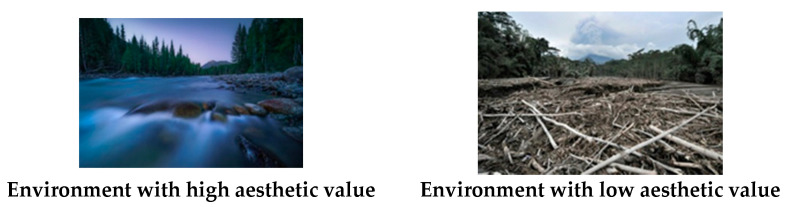
Examples of the environmental photographs as a priming stimulus in Experiment 1a.

In this study, the classification of the high and low aesthetic values of the environment was made mainly through the subjective dichotomous division and the aesthetic rating (7-point scale) of the environmental pictures by the participants in a pilot study. Finally, we chose environmental photographs with high and low aesthetic values and found a significant difference in the aesthetic rating scores. A total of 36 color photographs of the social and natural environments with high or low aesthetic values were selected from the public archive at http://baidu.com/ (accessed on 10 June 2020). The photographs were 500 × 300 pixels and processed using Adobe Photoshop. A separate group of 28 participants rated the photographs’ aesthetic quality and complexity on a 7-point scale. The results of the two sets of materials showed significant differences in aesthetic quality (5.74 ± 0.81; 2.80 ± 1.29, for environmental photographs with high and low esthetic values, respectively; [*t* (27) = 10.99, *p* < 0.05]), but no significant difference in terms of complexity ([*t* (27) = 0.88, *p* > 0.05]; high aesthetic value: 4.40 ± 1.27, low aesthetic value: 4.21 ± 1.42). The samples of the materials used in this study are shown in Figure 2.

**Figure 3:**
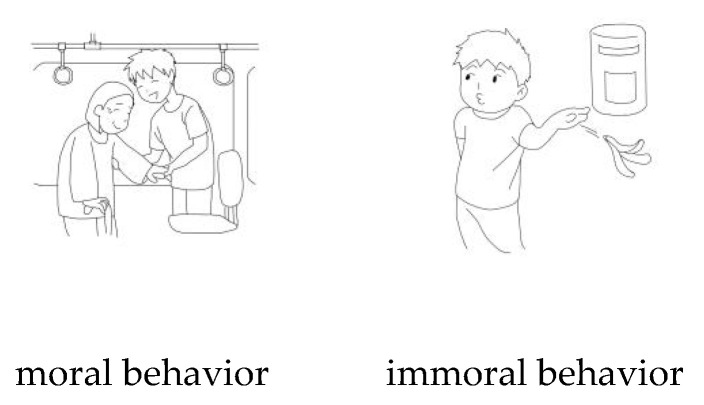
Example of behavioral materials used in Experiment 1.

Scene drawings for moral behavior were made in three sessions. First, we selected terms that describe moral and immoral behaviors through the semantic evaluation of college students. Second, we recruited students majoring in art to draw cartoon characters according to these terms. Third, we recruited 23 college students (14 females, 23.4 ± 1.67) to judge the moral degree, artistry, and complexity of the cartoon figures on a 7-point scale. Finally, 30 scene drawings of moral behaviors and immoral behaviors were chosen as materials for our moral judgment. Table 1 displays the mean scores of the different moral styles for different attributes. The results of the two sets of materials showed significant differences in terms of moral degree, but no significant difference in terms of complexity. The results of the artwork did not show a significant difference. Samples of the materials used in this experiment are illustrated in Figure 3. All experimental materials were adjusted to the same size using Adobe Photoshop.

##### Procedure

**Figure 4:**
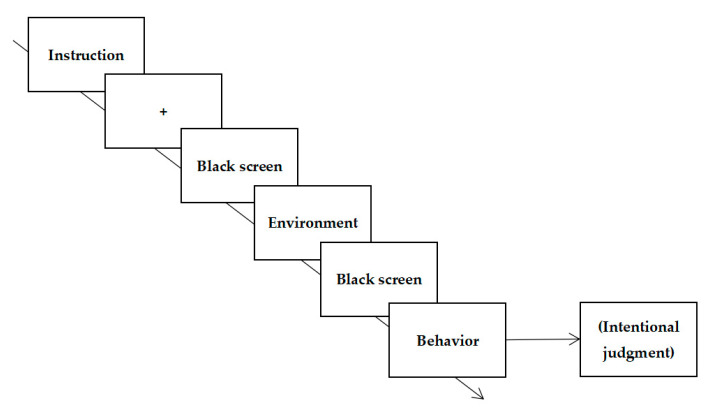
Example of event sequences in Experiment 1.

Each trial started with a fixation cross “+” for 500 ms followed by a 300 ms blank screen. Then, one of the environmental photographs was presented for 3000 ms, followed by a blank screen for 100 ms. Subsequently, a target image was presented until a key was pressed. Participants were instructed to report their possibility of engaging in these behaviors using subjective judgment on a scale ranging from 1 (not likely at all) to 9 (very likely). The experimental procedure is shown in Figure 4.

#### 2.1.2. Results and Discussion

The results showed that the data were normally distributed (*p* > 0.05). We computed the mean score for each participant’s behavioral intention and removed data with more than three standard deviations from the mean value as outliers (two data points were deleted). A 2 (types of environmental aesthetic value: high vs. low) × 2 (types of behavioral scene drawings: moral behavior vs. immoral behavior) repeated-measures analysis of variance (ANOVA) with subjects as the random effect was conducted on rating scores on behavioral intention.

**Figure 5:**
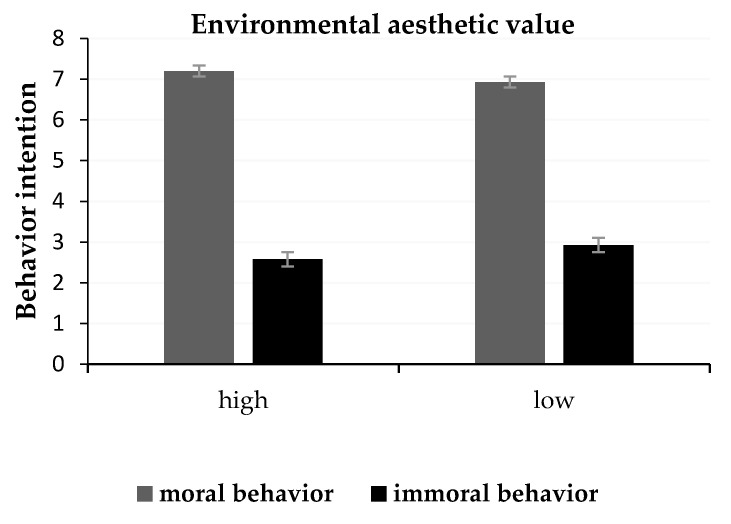
The scores of behavioral intentions in different environments.

Table 2 displays the mean scores of behavioral intentions in different environments. The results revealed that the main effect of the types of environmental aesthetic values was not significant, *F* (1, 22) = 0.16, *p* = 0.69 > 0.05, η^2^ = 0.57. The main effect of the types of behavioral scene drawings was significant, with *F* (1, 22) = 274.12, *p* < 0.001, η^2^ = 0.93 (see Table 2). The interaction between types of environmental aesthetic values and types of behavioral scene drawings was also significant, with *F* (1, 22) = 13.55, *p* = 0.001, η^2^ = 0.38 (see Figure 5). Moral behavior intention was slightly higher in an environment with a high aesthetic value than in an environment with a low aesthetic value, with *F* (1, 22) = 3.76, *p* = 0.065, η^2^ = 0.15. Immoral behavior intention was significantly lower in an environment with a high aesthetic value than in an environment with a low aesthetic value, *F* (1, 22) = 6.75, *p* = 0.016, η^2^ = 0.24.

The results of Experiment 1 showed that an environmental aesthetic value influences moral behavior intention and immoral behavior intention; additionally, it could trigger a higher intention for moral behavior and a lower intention for immoral behavior, compared to an environment with a low aesthetic value. These results are consistent with the hypothesis of Experiment 1a. The subjective assessment method was used to measure moral behavioral intention in Experiment 1. However, under experimental conditions, participants may hide their real behavioral intentions due to hypocrisy and the fear of being judged [31,35,36,37]. To explore and test the effect of an environmental aesthetic on behavioral intention for moral and immoral behavior, Experiment 1b used implicit measurements.

### 2.2. Experiment 1b

The objective of this experiment was to explore the association between environmental aesthetic value and behavior using IAT. In this study, participants were presented with a classification task in which they categorized environmental aesthetics using a standard IAT. If a participant performed the task quickly when an environment with a high aesthetic value was paired with moral behavior (as compared to the task in which an environment with a low aesthetic value was paired with moral behavior), it indicated a positive implicit relation between environmental aesthetic value and behavior.

#### 2.2.1. Method

##### Participants

A total of 34 college participants aged from 18 to 24 years (20 females; *M* age = 20.85 years, *SD* = 0.85), who did not participate in Experiment 1a, were recruited and paid for their participation. All participants had normal or corrected normal vision and normal color vision. We used G * Power 3.1 to estimate the power (1-*β* = 0.81) and effect size (d = 0.64). The participants signed an informed consent form, and the experiment was approved by the Institute Ethics Committee of South China Normal University.

##### Materials

The scene drawings for moral behavior in Experiment 1b were similar to those in Experiment 1a. The environmental pictures were chosen according to the procedure of Experiment 1. All pictures were 500 × 300 pixels and adjusted using Adobe Photoshop. The rating results of the two sets of materials showed a significant difference in terms of beauty quality, with *t* (27) = 10.35, *p* = 0.001 (photographs of the high aesthetic value, 5.72 ± 0.83; photographs of the low aesthetic value, 2.93 ± 1.34) and no significant difference in terms of complexity, with *t* (27) = 0.19, *p* = 0.84 (high aesthetic value: 4.20 ± 1.23; low aesthetic value: 4.16 ± 1.45). Participants completed an IAT task that measured their implicit associations between the environment and their behaviors.

##### Procedure

Each participant completed a total of seven classification tasks: (1) single categorization for the target (environmental photograph with a high or low aesthetic value; 18 trials); (2) single categorization for the implicit association (moral /immoral behavior; 24 trials); (3) combined categorization task-practice and data collection trials (environmental photograph with a high aesthetic value + moral behavior/environmental photograph with a low aesthetic value + immoral behavior; 42 trials); (4) the same as (3); (5) single categorization for the target concept (same as (2)) but with reversal of the side of the screen where the category into which the picture needed to be categorized was presented (18 trials); (6) combined categorization task-practice and data collection trials (same as (3)) but reversed categorization of target categories (environmental photograph with a low aesthetic value + moral behavior/environmental photograph with a high aesthetic value + immoral behavior; 42 trials); (7) same as (6). Only data from tasks (3), (4), (6), and (7) were used for the analysis.

Participants completed an IAT task measuring the implicit associations between environments and behaviors [38]. Subjects responded to the categorization task by pressing either the “E” key with the left-hand finger or the “I” key on the numeric keypad with the right-hand finger. The meaning of “E” or “I” were shown in Table 3.

#### 2.2.2. Results and Discussion

##### Data Reduction

We applied a data reduction procedure: the first trial of each experimental task was removed before the analysis, and a latency longer than 10,000 ms and shorter than 300 ms was also removed [38]. In this study, no data were excluded from the analysis because of an error rate lower than 20%. We checked the data and determined that they were not normally distributed (*p* < 0.05); therefore, non-parametric tests were used to compare differences in means.

We compared the categorization of environments paired with moral and immoral behaviors. Table 4 displays the mean accuracy (ACC) and reaction time (RT) under different conditions. The results provide implicit attitudes toward these two categories.

Participants had significantly shorter reaction times when the environments with a high aesthetic value were paired with moral behaviors, as compared to environments with a low aesthetic value paired with moral behaviors (*p* < 0.05). Quicker RT for an environment with a high aesthetic value, together with moral behaviors, indicated the existence of an implicit association between the two.

The result showed a higher accuracy when the environments with a high aesthetic value were paired with moral behaviors, as compared to the environments with a low aesthetic that were paired with immoral behaviors (*p* < 0.05).

##### IAT Effect

Table 5 displays the mean RT for the different parts of the joint discrimination task. Drawing on the results of different parts of the joint discrimination task, we calculated d1 and d2. Finally, d1 = 1.23, d2 = 1.25, d = (d1 + d2)/2 = 1.24.

Compared to the RT of the two joint tasks, the results showed that the RT of the initial joint task was significantly longer than that of the reverse joint one (*t* = 9.49, *p* < 0.01). Previous research suggested that the difference between the two groups of experimental effects is stronger when the d-score is greater than or equal to 0.8. In experiment 1b, the d-scores were 1.24, and the result showed a significant IAT effect.

The results of Experiment 1b demonstrated the existence of implicit relations between the environments with high aesthetic values and moral behaviors and between the environments with low aesthetic values and immoral behaviors. The result supported our hypothesis, which informs an association between the environments with high aesthetic values and moral behaviors in evolution, eventually leading to a higher intention for moral behavior compared to the environments with low aesthetic values.

In general, these findings are consistent with the idea that the environment is related to behavior [1,2,10,23]. Previous research found that emotion influences moral and immoral behaviors; positive emotions lead to an increase in moral behavior, and negative emotions lead to an increase in immoral behavior [13,19,39]. Therefore, for the changes in behavioral intention for moral and immoral behaviors, we found that positive emotion activated by the environment with a high aesthetic value and negative emotion activated by the environment with a low aesthetic value can lead to a difference in behavioral intention.

In addition, regarding the effect of the environmental aesthetic value on the behavioral intention for moral and immoral behavior, previous research has suggested that behavior is related to moral cognition [40]. Villegas and Vargas-Trujillo (2015) showed that moral judgment has a consistent effect on behavior [41]. Therefore, for the effect of environments on the intention to behave differently, we proposed a possible explanation: that different aesthetic values influence moral judgment for behaviors. Experiment 2 tested this hypothesis.

Therefore, Experiment 2 first tested the positive relationship between behavioral intention and moral judgment in Experiment 2a; then, in Experiment 2b, it explored the effect of the environmental aesthetic value on moral judgment and whether the change in moral judgment is the same as the intention for behavior in different environments. We proposed that participants may have a higher score of moral judgment for moral behaviors and a lower score of moral judgment for immoral behaviors in an environment with a high aesthetic value, compared to an environment with a low aesthetic value (that is, there is a positive relationship between moral judgment and behavioral intention).

## 3. Experiment 2

### 3.1. Experiment 2a

Experiment 2a mainly tested the positive relationship between moral judgment and behavioral intention: in other words, whether the changes in moral judgment were the same as the changes in behavioral intention.

#### 3.1.1. Method

##### Design

Experiment 2a was a single-factor, within-subject experiment design. The independent variable was the type of behavioral scene drawings (moral vs. immoral). Every participant was instructed to make moral judgments and judgments of behavioral intention for the same behavioral scene drawings. The dependent variables were the rating scores for behavioral intention and moral judgment.

##### Participants

A new group of 62 college participants aged 18–24 years (11 males; *M* age = 18.40, *SD* = 1.03) were recruited and compensated for their participation. We used G * Power 3.1 to estimate the power (1-*β* = 0.97) and effect size (d = 0.47). All participants had normal or corrected normal vision and normal color vision. They signed an informed consent form. The study was approved by the Institute Ethics Committee of South China Normal University.

##### Materials

The materials of the behavioral scene drawings in Experiment 2a were the same as those in Experiments 1a and 1b (see Figure 3).

##### Procedure

The stimuli were presented to the participants, who were instructed to make a moral judgment on a scale ranging from 1 (very immoral) to 9 (very moral). Finally, participants were instructed to report the possibility that they would engage in these behaviors using subjective judgment on a scale ranging from 1 (not likely at all) to 9 (very likely).

#### 3.1.2. Results and Discussion

We checked the data, determined that they were normally distributed (*p* < 0.05), and logarithmically transformed them into positively distributed data. Then, we performed a correlation analysis, the results of which are presented in Table 6.

##### Regression Relation Testing

The mean scores of moral judgments predicted those of moral behavior intention (*β* = 0.33, R^2^ = 0.11, y = 0.33x + 5.62, *p* = 0.01). The mean scores of immoral judgments predicted the scores for immoral behavior intention (*β* = 0.62, R^2^ = 0.39, y = 0.62x + 1.65, *p* < 0.001).

The results showed that moral judgment is related to behavioral intention for moral or immoral behavior, which is consistent with the findings of a prior study [17]. These also support the relationship among different factors of moral decisions. Experiment 2b mainly tested the effect of an environmental aesthetic value on moral judgment and explored the mechanisms by which behavioral intention changes in environments with different aesthetic values.

### 3.2. Experiment 2b

#### 3.2.1. Method

##### Design

Experiment 2b was a 2 (types of environmental aesthetic value: high vs. low) × 2 (types of behavioral scene drawings: moral vs. immoral) within-subject experimental design. The participants evaluated morality for the same behaviors in the same environment. The dependent variable was the rating score for moral judgment.

##### Participants

A new group of 38 college students aged 18–24 years (24 females; *M* age = 22.03, *SD* = 1.15) were recruited and compensated for their participation. We used G * Power 3.1 to estimate the power (1-*β* = 0.95) and the effect size (d = 0.24). All participants had normal or corrected normal vision and normal color vision. They signed an informed consent form. The study was approved by the Institute Ethics Committee of South China Normal University.

##### Materials

The materials of environmental photography and behavioral scene drawings in Experiment 2b were the same as in Experiment 1a.

##### Procedure

**Figure 6:**
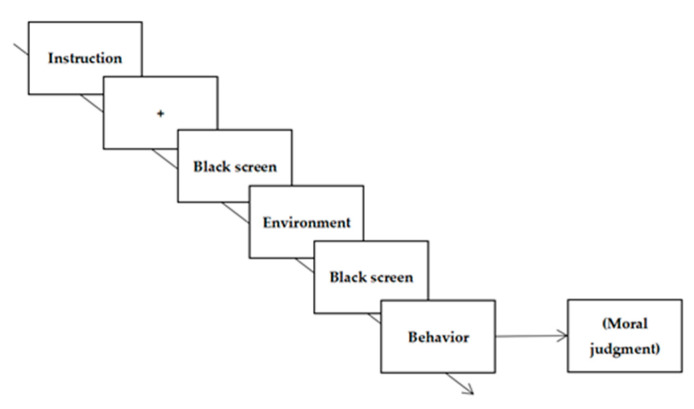
Example of event sequences on the moral judgment task in Experiment 2b.

The procedure of Experiment 2b was the same as that used in Experiment 1a. However, Experiment 2b required participants to make moral judgments regarding behaviors. Participants were instructed to make moral judgments on a scale ranging from 1 (very immoral) to 9 (very moral) when the target behavior was presented (see Figure 6).

#### 3.2.2. Results and Discussion

We checked the data and determined that they were normally distributed (*p* > 0.05). We then conducted 2 (types of behavioral scene drawings: moral behavior vs. immoral behavior) × 2 (types of environmental photograph: high aesthetic value vs. low aesthetic value) repeated-measures ANOVA. The dependent variable was the rating score of moral judgment. The mean rating scores for moral judgment are shown in Table 7.

**Figure 7:**
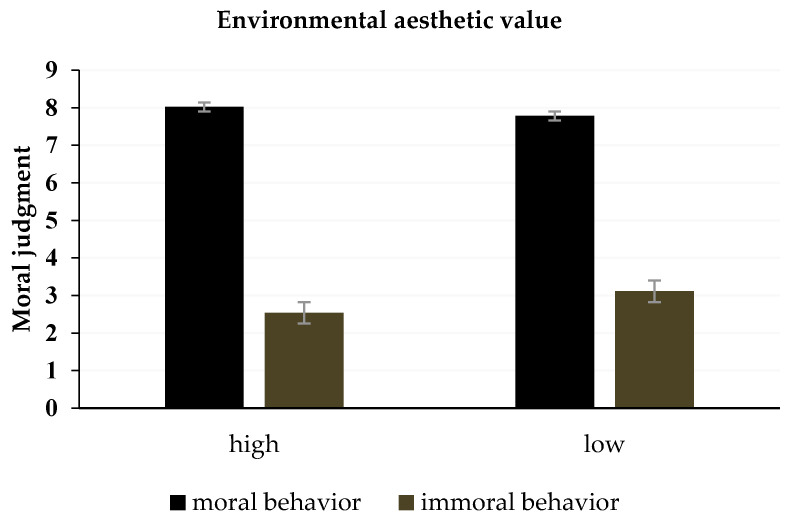
The interaction effect between environmental style and behavioral style on moral judgment.

The results revealed that the main effect of the environmental style was significant, with *F* (1,37) = 4.69, *p* = 0.037, η^2^ = 0.11. The condition of high aesthetic value was perceived as having lower morality than the condition of low aesthetic value. The main effect of the behavioral style was significant, with *F* (1,37) = 644.81, *p* < 0.001, η^2^ = 0.95. The interaction between environmental style and behavioral style was also significant, with *F* (1,37) = 48.94, *p* < 0.001, η^2^ = 0.57. The simple effect test showed that moral behavior was evaluated as having higher morality in an environment with a high aesthetic value compared to an environment with a low aesthetic value, with *t* (37) = 2.49, *p* = 0.017, 95% CI = [0.047, 0.45]. Immoral behavior was significantly higher in an environment with a high aesthetic value than in an environment with a low aesthetic value, with *t* (37) = −6.46, *p* < 0.001, 95% CI = [−0.78, −0.39] (see Figure 7).

The results of Experiment 2 found that individuals in an environment with a high aesthetic value reported higher morality for moral behavior and lower morality for immoral behavior, compared to an environment with a low aesthetic value, which supports the hypothesis of Experiment 2. These results showed that the environment could affect moral judgment and confirmed the role of the environment in it. Based on the results of Experiments 1 and 2, we found a positive relationship between moral judgment and the intention to behave. When participants reported higher morality for moral behavior in an environment with a high aesthetic value, they also reported higher intention for these behaviors, compared to an environment with a low aesthetic value. Conversely, when participants reported lower morality for immoral behavior in an environment with a low aesthetic value, they reported lower immoral behavior intention, compared to a high aesthetic value.

## 4. Discussion

Experiment 1 explored the relationship between environmental aesthetic values and moral and immoral behavioral intentions. The results showed that an environment with a high aesthetic value leads to a higher behavioral intention for moral behavior, and a low aesthetic value leads to a higher behavioral intention for immoral behavior. The results of Experiment 1 confirmed that the environmental aesthetic value can increase moral behavioral intention, which is in line with the effect of beauty on pro-social behavior [13]. Moral behavior and immoral behavior are positively related to moral judgment [19,20]; thus, Experiment 2 tested the role of moral judgment in the relationship between environmental aesthetic value and behavioral intention. The results showed that individuals judge higher morality for moral behavior and lower morality for immoral behavior in an environment with a high aesthetic value, compared to an environment with a low aesthetic value. These results showed the same direction of change between moral judgment and behavioral intention. The findings support the results of previous studies, which demonstrate that the environment can influence cognition toward moral behavior [24,25,34]. They also support our hypothesis, that changes in moral judgment affect behavioral intention in different environments.

Previous researchers have pointed to the emotion hypothesis to explain the effect of environmental beauty on pro-social behaviors [13]. Different environments trigger individuals’ emotions, which influence moral behavior [8,13,42]. Therefore, the effects of environmental aesthetic values on behavioral intention might be emotions that are triggered by different environment conditions. Emotions also affect moral judgment. Strohminger, Lewis, and Meyer (2017) found that mirth increased permissiveness for utilitarian solutions to moral dilemmas [32], and disgust led participants to make harsh judgments for immoral behaviors [43]. In other research, beauty, or the lack thereof, was found to activate positive and negative emotions, respectively [44]. People who experience positive emotions exhibit significantly more unusual cognition [45], such as being more flexible and tolerant [46,47,48,49]. However, the results of Experiment 2 did not find a lower moral score for immoral behavior in an environment with a low aesthetic value, compared to a high aesthetic value environment. This result is in contrast with the findings of previous research [26,27,50]. Context could affect aesthetic judgment, which might generate different results [51,52,53,54,55,56]. When the context stimulus was formally similar to one of the two artworks used in comparison but aesthetically slightly inferior to it, an assimilation effect was observed, which showed that the target assimilated the context [57,58,59]. In contrast, when the context was similar to the target but definitely of inferior aesthetic quality, a contrast effect was observed, which showed that the judgment of the target was pushed down (positive context) or pushed up (negative context) by the context [55,60,61,62]. People always make judgments that equate one standard of judgment and perception perception [63,64]. The physical environment is a factor. Meanwhile moral behavior has a high aesthetic value, and immoral behavior has a low aesthetic value [65]. Therefore, for the different results of Experiment 2, we suggest that a strong difference in aesthetic value occurs for immoral behavior, When the environment has a high aesthetic value. Finally, an environment with a high aesthetic value leads to contrasting effects for immoral behavior and assimilation effects for moral behavior, compared to an environment with a low aesthetic value.

In addition, several environmental psychology theories, such as emotional arousal theory and environmental load theory, have been put forward to explain the relation between the environment and behavior. The former suggests that environmental stimuli affect individuals’ level of emotional arousal, thereby triggering or inhibiting certain behaviors [66]. The latter suggests that individuals have very limited processing of external information and a limited capacity for input from external stimuli; further, environments contain different amounts of information and those with more information can lead to cognitive overload and affect individuals’ behavior [66] Regarding the effect of environmental aesthetic value on moral behavior, Zhang et al. (2014) preferred arousal theory [13], which did not explain the results of the present research (a higher score of moral judgment for immoral behavior in an environment with a low aesthetic value than in an environment with a high aesthetic value). Thus, we proposed a new hypothesis to explain the relation between the environment and behavior, by which different environments are related to different behaviors, which influence behavioral decisions. We argue that there is a matching relationship between the two and that this leads to changes in individuals’ moral behavior intention and moral judgment in environments with different aesthetic values. In a certain environment, matching behaviors are more likely to emerge, and are also evaluated more leniently. Thus, moral behavior intention was higher, and it had a more lenient judgment in an environment with a high aesthetic value compared to an environment with a low aesthetic value. Immoral behavior intention and moral judgment were the opposite in an environment with a different aesthetic value. Immoral behavior intention was lower, and moral judgment was more lenient in an environment with a low aesthetic value than in one with a high aesthetic value.

### Limitations

The environment plays an important role in human life [13,42]. Our research found that beauty has an effect on the natural and social environments. However, we did not take into account the participants’ basic moral or sociodemographic characteristics, which possibly had some influence on the results. For example, if all participants were at extremely high (or very low) moral levels, the floor or ceiling effect might occur on the participants’ reported moral behavioral intentions, which would possibly confound the influence of the environmental aesthetic value on moral behavioral intentions. In the present study, we only conducted a comparison between the high and low aesthetic value environments, not with the corresponding baseline, i.e., the neutral environment. Although the difference between the two was demonstrated, the difference between the high or low aesthetic value and the neutral (baseline) is unclear. This would lead to a limited effect of high aesthetic value enhancement, which is only compared to low aesthetic value environments.

In environmental psychology, many researchers have focused only on the effects of the natural environment. However, everyone relates to the social environment and accepts its effects; therefore, efforts should be made to explore the role of an aesthetic social environment in the future.

In this experiment, Experiment 1 and Experiment 2 both had small samples that may lack some external validity. The results of this study support the view that aestheticians can shape and change human behavior through the environment. The broken windows theory determines the effect of a disorganized environment on moral behavior; in later studies, researchers and government officers empirically verified the power of the environment [1,67]. Thus, the results of the present research could be used in future aesthetics education and moral education, and provide a new perspective on the prevention of immoral behaviors.

## 5. Conclusions

In summary, the results of this study reveal that participants’ moral judgment can be influenced by environments with different aesthetic values, which in turn influence behavioral intentions. The current research also provides a new viewpoint for understanding the relationship between the environment and behavior. The results of this study can help society positively influence people’s social behavior through the environment as an objective setting. Whether it is in the family, school, or other public places, by shaping this beautiful environment, people’s esthetic and moral education can be influenced.

## Figures and Tables

**Table 1:** The mean rating scores of different behavior styles in different attributes.

Moral Style	The Attributes		
	Morality	Artistry	Complexity
Moral	6.02 ± 0.74	4.43 ± 0.79	3.98 ± 0.99
immoral	2.05 ± 0.53	4.18 ± 0.40	3.88 ± 0.44
*P*	<0.001	>0.05	>0.05

**Table 2:** Mean rating score of behavioral intentions in different environments (M ± SD) in Experiment 1a.

Behavior	Environmental Aesthetic Value	The Score of Behavioral Intention	
		M	SD
Moral	High	7.20	0.87
Moral	Low	6.93	0.93
Immoral	High	2.58	0.82
Immoral	Low	2.93	0.64

**Table 3:** The meaning of “E” and “I” in every task.

Task	“E” Key	“I” Key
1	High aesthetic value	Low aesthetic value
2	Moral	Immoral
3	High/Moral	Low/Immoral
4	High/Moral	Low/Immoral
5	Immoral	Moral
6	High/Immoral	Low/Moral
7	High/Immoral	Low/Moral

**Table 4:** The mean ACC and reaction time in different conditions.

Style	ACC		RT	
	M	SD	M	SD
Compatibility	0.98	0.021	857.15	234.08
No-compatibility	0.94	0.046	1586.81	625.90

**Table 5:** The mean RT and SD in different parts of the joint discrimination task.

	RT	
	M(ms)	SD
Third part	915.33	274.13
Fourth part	798.96	206.32
Sixth part	1724.93	685.95
Seventh part	1413.70	504.92

**Table 6:** The correlation between moral judgment and behavioral intention.

	The Moral Judgment of Moral Behavior	The Moral Judgment of Immoral Behavior
Moral behavior intention	0.33 **	
Immoral behavior intention		0.62 **

** stands for <0.01.

**Table 7:** Mean rating scores of moral judgments in different environments (M ± SD) in Experiment 2b.

Behavior	Environmental Aesthetic Value	The Indirect Moral Judgment	
		M	SD
Moral	High	8.02	0.89
Moral	Low	7.78	0.89
immoral	High	2.54	0.77
immoral	Low	3.11	0.59

## Data Availability

The data presented in this study are available on request from the corresponding author. The data are not publicly available due to restrictions e.g., privacy or ethical.
